# Hunting behavior of a solitary sailfish *Istiophorus platypterus* and estimated energy gain after prey capture

**DOI:** 10.1038/s41598-023-28748-0

**Published:** 2023-01-27

**Authors:** Ryan K. Logan, Sarah M. Luongo, Jeremy J. Vaudo, Bradley M. Wetherbee, Mahmood S. Shivji

**Affiliations:** 1 Guy Harvey Research Institute, Nova Southeastern University, Dania Beach, FL USA; 2 Department of Biological Sciences, Florida International University, North Miami, FL USA; 3 Department of Biological Sciences, University of Rhode Island, Kingston, RI USA

**Keywords:** Ecology, Physiology, Bioenergetics, Computer modelling, Time series, Behavioural ecology, Biooceanography, Ecophysiology, Marine biology

## Abstract

Foraging behavior and interaction with prey is an integral component of the ecological niche of predators but is inherently difficult to observe for highly mobile animals in the marine environment. Billfishes have been described as energy speculators, expending a large amount of energy foraging, expecting to offset high costs with periodic high energetic gain. Surface-based group feeding of sailfish, *Istiophorus platypterus,* is commonly observed, yet sailfish are believed to be largely solitary roaming predators with high metabolic requirements, suggesting that individual foraging also represents a major component of predator–prey interactions. Here, we use biologging data and video to examine daily activity levels and foraging behavior, estimate metabolic costs, and document a solitary predation event for a 40 kg sailfish. We estimate a median active metabolic rate of 218.9 ± 70.5 mgO_2_ kg^−1^ h^−1^ which increased to 518.8 ± 586.3 mgO_2_ kg^−1^ h^−1^ during prey pursuit. Assuming a successful predation, we estimate a daily net energy gain of 2.4 MJ (5.1 MJ acquired, 2.7 MJ expended), supporting the energy speculator model. While group hunting may be a common activity used by sailfish to acquire energy, our calculations indicate that opportunistic individual foraging events offer a net energy return that contributes to the fitness of these highly mobile predators.

## Introduction

Predator–prey interaction is a cornerstone of ecology, intrinsically linked to individual fitness and population level dynamics of both predators and prey, and ultimately relates to the evolutionary success of populations^1,2^. Foraging behavior and energetic gains and losses associated with foraging, predation and consumption impinge directly on the physiology and behavior of all animals^2,3^. For many large pelagic marine predators, the rarity of observations of predatory events and challenges of documenting hunting behavior have hindered our understanding of behavioral strategies, trophic relationships and associated energetics in marine ecosystems^4^.

Istiophorid billfishes (marlins, spearfishes, sailfish) are known for their unique morphology, power, and high-speed predatory potential. In sailfish (*Istiophorus platypterus*), an epipelagic predator inhabiting tropical to subtropical waters worldwide^5^, group hunting behavior is well documented and involves multiple individuals herding a school of prey fish (i.e. bait ball) toward the water’s surface. Individual sailfish enter the school and laterally slash their bill in an attempt stun/kill prey for consumption^6–8^. This tactic is facilitated by morphological adaptations including the bill, a streamlined body shape, enlarged dorsal fin that acts to stabilize the sailfish as it slashes the bill, and a caudal fin with a high aspect ratio enabling bursts of speed of up to 8.8 ms^−1^ during bait-ball interactions^9^.

Outside of bait-ball hunting aggregations, however, sailfish are believed to be solitary roaming predators. Because of the difficulty of maintaining sailfish in captivity, energetic requirements have not been directly measured for adults [but see^10^], yet due to various life history and morphological traits, sailfish are presumed to have a high metabolic rate^11–13^. As such, in addition to bait-ball hunting events, solitary sailfish likely need to capitalize on encounters with prey to support this high metabolic rate. However, due to an elusive pelagic lifestyle, individual sailfish hunting behavior has not been documented and energetic relationships of such events have not been investigated. Recently, sailfish have been described as lateralized predators, preferring to attack from one side (right or left) of their prey, depending on the individual^8^. This is theorized to have evolved via group hunting, where multiple sailfish take turns attacking a bait-ball, preventing the prey from learning which side the attack may come from^8^. However, in one-on-one predator–prey interactions, lateralization could be costly to the predator’s hunting success because it would increase the predictability of where an attack will come from^14,15^. Lack of information on hunting behavior and energetics of such events, which have direct bearing on ecological interactions of top predators, results in a limited understanding of their role in oceanic ecosystems and overall fitness^16^.

Knowledge of daily activity levels and energy dynamics of hunting behavior and foraging events in billfishes and other top marine predators will improve our understanding of behavioral alterations associated with changing environmental conditions, such as warming and deoxygenation^17^. Because activity level has a major impact on an animal’s energy budget, there is a need for estimates of active metabolism of large aquatic predators to inform future energetic and trophic models. Here, using an animal-borne datalogger with video, we report on the daily activity of an individual sailfish in the Eastern Tropical Pacific over a 24 h period. We describe an observation of a foraging event, the lateralization of the strikes during the event, and place these events in the broader context of the daily activities of this individual to estimate the daily net energetic benefit of the predation event.

## Results and discussion

**Figure 1:**
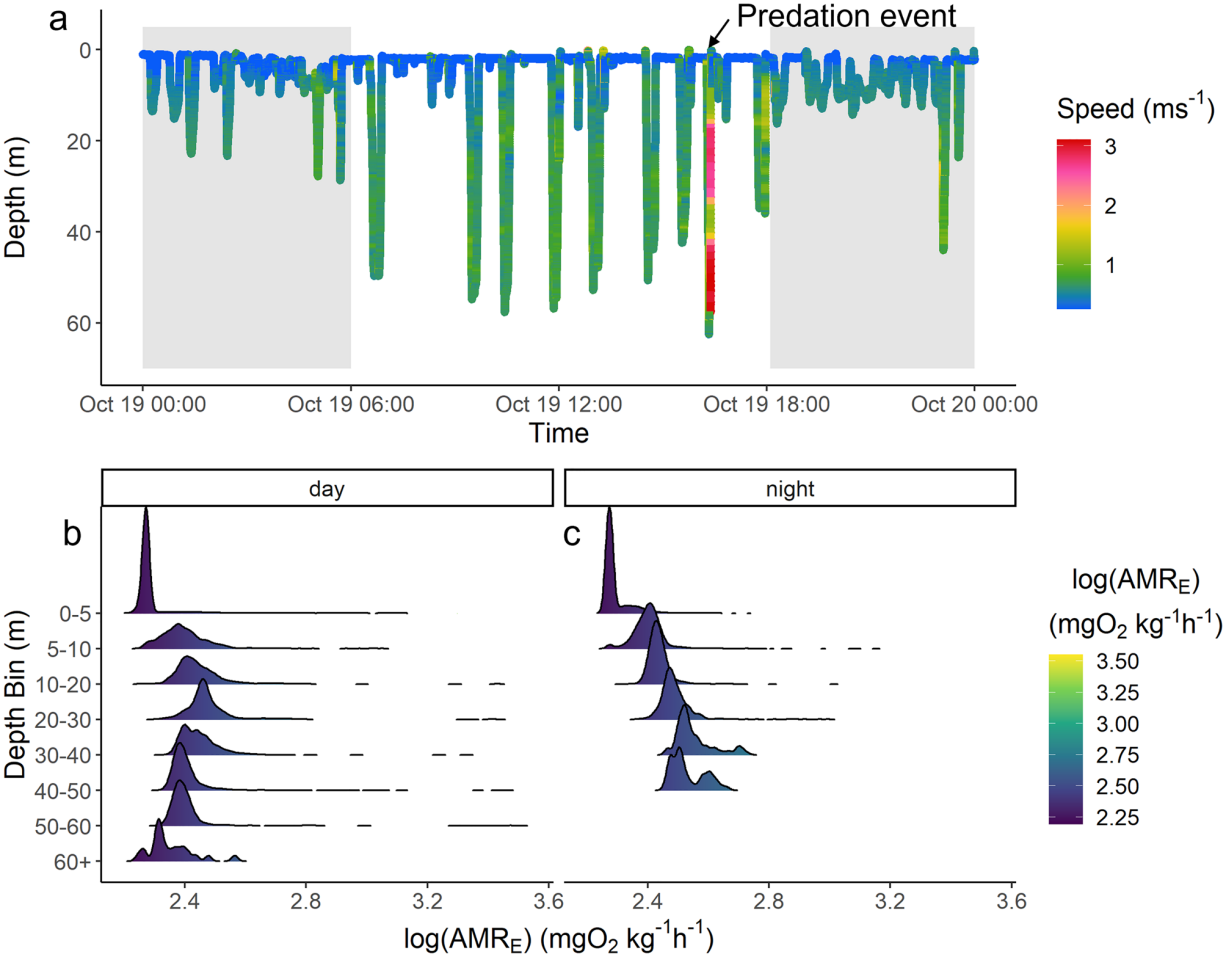
Depth trace over the course of the 24 h monitoring period in (**a**), and binned depth density histograms of the log transformed median estimated active metabolic rate (AMR_E_; mgO_2_ kg^−1^ h^−1^) for day and night periods in (**b**) and (**c**), respectively. In (**a**), color of the depth trace indicates speed (ms^−1^), and shaded regions represent night hours. The dive in which the predation event took place is indicated with an arrow.

We used a custom designed biologging tag package with onboard video to describe a 3D high-resolution pursuit between a solitary sailfish and an individual small tuna in open water, representing the first time such an interaction has been documented. The sailfish was tagged at 09:53 on 18 October 2019, and the tag package remained attached to the sailfish for 67 h. However, analyses here are limited to the 24 h period in which the predation event took place (19 October–20 October; ~ 14 h after tagging and ~ 9 h after post-release recovery^18^) because this coincides with the time period the video camera was recording during daylight hours (on at 0600, off at 1800, sunrise and sunset, respectively) enabling us to ground-truth acceleration signals. Biologging data and accompanying video show the sailfish performing oscillatory dives between the surface and depths of 40–50 m during daylight hours. At night, fewer dives were performed and the sailfish generally remained within the top 10–20 m of the water column (Fig. 1a), leading to a greater range of temperatures experienced during the day (day 20.9–27.9 °C; night 26.5–28.2 °C). Due to the temperature dependence of the estimated active metabolic rate (AMR_E_), the cooler temperatures at depth led to a reduced AMR_E_ during daylight hours (212.9 ± 89.1 mgO_2_ kg^−1^ h^−1^) compared to night (224.7 ± 44.4 mgO_2_ kg^−1^ h^−1^). Additionally, AMR_E_ initially increases with depth due to increased swim speeds during diving (Fig. 1b), until the thermocline is reached in the 30–40 m depth bin, at which point AMR_E_ decreases with further increased depth (Fig. 1b, c). However, due to thermal inertia of large-bodied fishes^19–21^, it is possible that the sailfish’s body retained heat during the short (14.7 ± 1.7 min) excursions below the thermocline and did not drop to ambient temperature. As such, the metabolic rate calculated at depth may be underestimated with the temperature correction performed here. For example, during the dive in which the predation event occurred (Fig. 1; Table 1), if body temperature was assumed equivalent to surface temperature throughout the dive, estimated metabolic rates would increase by 18% compared to if the metabolic rates were temperature corrected according to the tag's external temperature reading (Table S2). Yet, because the majority (> 90%) of time over the 24 h was spent above the thermocline, the temperature correction has little impact on the daily calculated AMR_E_ and subsequent energy expenditure (< 1% difference; Table S3).

**Table 1:** Summary statistics for dives performed by the sailfish during daylight hours compared to the dive in which the predation event took place. Values are presented as mean ± SD where applicable.

	Daytime dives (n = 8)	Pursuit dive
Duration (min)	14.7 ± 1.7	6.7
Max depth (m)	50 ± 7.5	62.4
Descent rate (ms^−1^)	0.18 ± 0.03	0.24 ± 0.21
Ascent rate (ms^−1^)	0.17 ± 0.03	1.3 ± 0.43
Max AMR_E_ (mgO_2_ kg^−1^ h^−1^)	498.2 ± 92.2	3283.8
Energy used (MJ)	0.03 ± 0.003	0.04

The sailfish exhibited greatly reduced tailbeat activity and swimming speeds (≤ 0.25 ms^−1^; 0.14 body lengths [BL] s^−1^) when near the surface (Fig. 1), characteristic of basking behavior exhibited by swordfish and other istiophorid billfishes^22,23^. Basking is believed to serve thermoregulatory purposes^23^, but would also serve to reduce energy expenditure for billfishes facilitated by their swim bladders^24^. Indeed, basking behavior observed here led to a significant reduction in AMR_E_ (186.6 ± 3.1 mgO_2_ kg^−1^ h^−1^; T_37520_ = − 158.3, *p* < 0.001), when compared to active swimming behavior with strong and sustained tailbeats during dives (261.1 ± 91.1 mgO_2_ kg^−1^ h^−1^; mean swimming speed 0.56 ± 0.2 ms^−1^; 0.3 ± 0.1 BL s^−1^).

**Figure 2:**
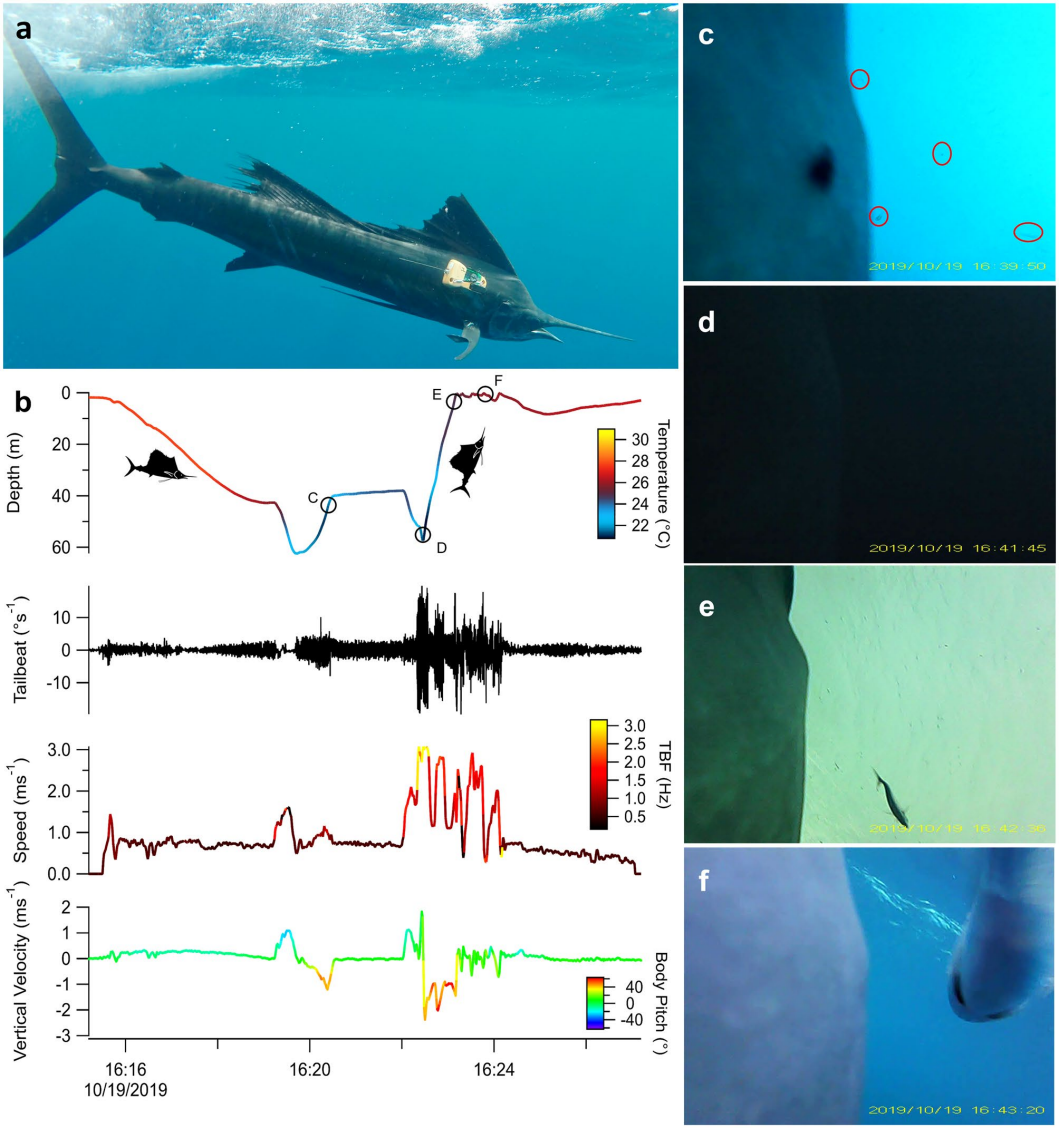
Sailfish activity before, during and after the predation event. (**a**) Biologging float package attached to sailfish. (**b**) Depth, temperature, tailbeats (°sec^−1^), speed (ms^−1^), tailbeat frequency (TBF; Hz), vertical velocity (ms^−1^) and body pitch angle (°) of the dive in which the event occurred. The timing and depth associated with each image (**c**–**f**) are identified by circles on the depth profile in (**b**). (**c**) The sailfish ascends from 60 m and encounters multiple potential prey items, outlined in red. (**d**) The available light is notably low at this depth and decreases rapidly with depth to almost zero light. (**e**) First observation of the prey. (**f**) Prey possibly attempting to ‘hide’ from the sailfish by swimming very close to it during the pursuit.

The dive in which the predation event took place occurred roughly 31 h into the 67 h that the tag package remained attached to the sailfish (Fig. 2a). At 16:15 19 October 2019, the sailfish dove from the surface to a depth of 62.4 m with a mean (± SD) vertical velocity (VV) of 0.24 ± 0.21 ms^−1^, where it remained for a short period before ascending to ~ 40 m (Fig. 2b). During the ascent, multiple possible prey items are seen in the video (Fig. 2c), and there was a brief increase in speed and tailbeat frequency (TBF), before the fish’s depth leveled off for ~ 2 min. The fish then dove again to 57.5 m, where visible light almost completely attenuated (Fig. 2d), and a change in locomotory mode from slow and steady swimming to rapid and forceful tailbeats occurred, beginning a rapid ascent (VV = 1.3 ± 0.43 ms^−1^), with speeds reaching 3.1 ms^−1^ (1.7 BL s^−1^), and a body pitch of 54.6 ± 16.1° (maximum of 77.6°; Fig. 2b). It should be noted that due to the rapid ascent, the temperature readout of the tag lagged behind true ambient temperature (e.g., temperature of the descent compared to temperature of the ascent; Fig. 2b). Summary statistics for the dive in which the predation event took place are compared to all other daytime dives (Table 1).

**Figure 3:**
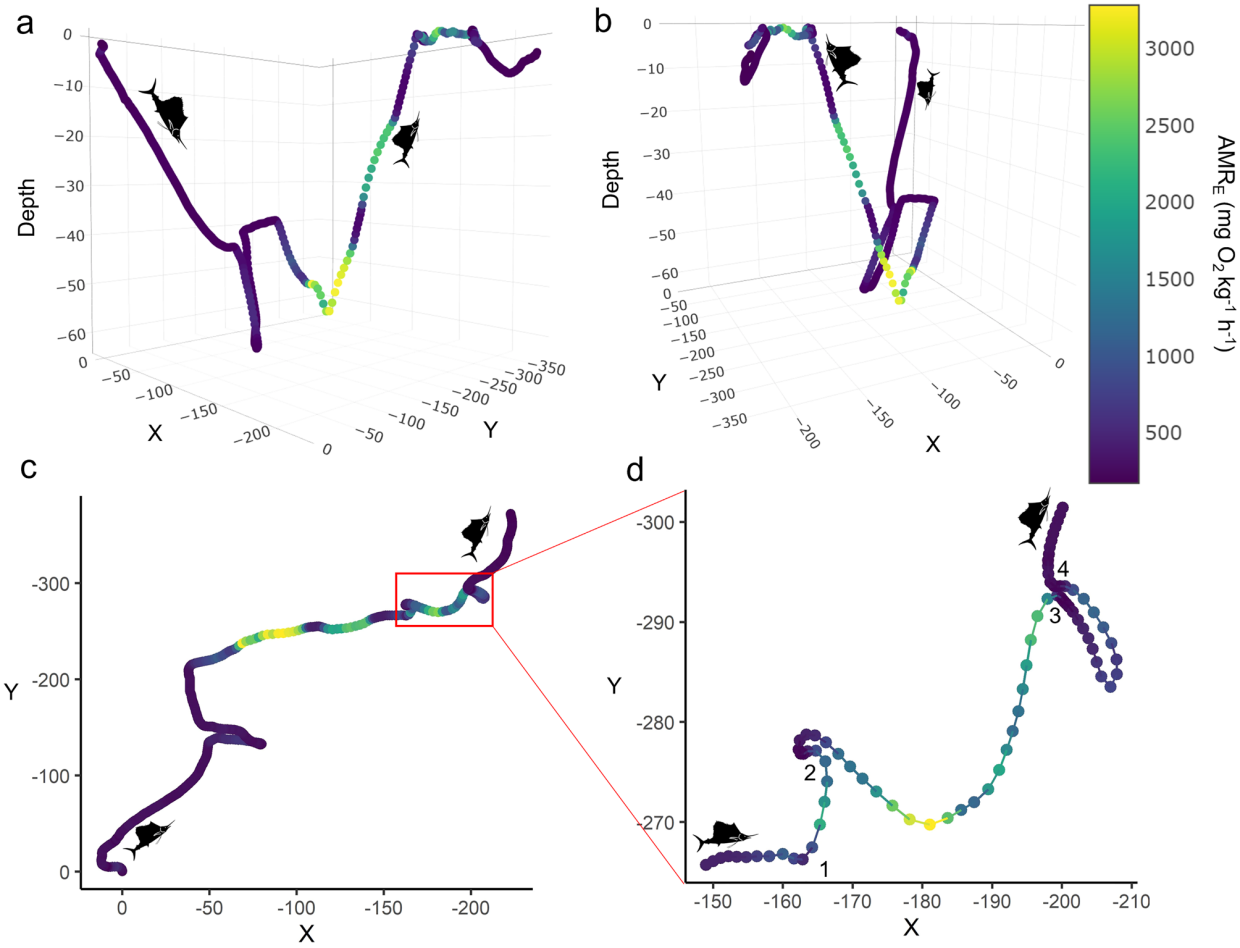
Reconstructed track of the dive in which the predation event took place, colored by the estimated active metabolic rate (AMR_E_; mgO_2_ kg^−1^ h^−1^). (**a**) and (**b**) show different perspectives of the depth profile and associated changes in direction, while (**c**) and (**d**) are overhead views, with (**d**) being zoomed in on the pursuit portion at the surface. The numbers displayed in (**d**) represent the approximate location of the sequential capture attempts displayed in Fig. 4, and as seen in the supplemental video. Sailfish silhouettes indicate direction of travel.

The prey that was pursued during the predation event first became visible in the video when the sailfish reached the surface (Fig. 2e). The sailfish made several attempts to capture the prey, often breaking the surface of the water (supplemental video). From the video, the prey appeared to be a frigate or bullet tuna (*Auxis thazard brachydorax* or *A. rochei eudorax*), both of which are common in the region and known sailfish prey^5,25^. During the rapid ascent and while at the surface, TBF and swimming speed remained high (1.6 ± 0.7 Hz and 1.7 ± 0.84 ms^−1^, respectively, maximum of 2.92 ms^−1^). At the surface, there were frequent changes in heading and the tuna appeared in the video several times (Fig. 2b; Fig. 3d; supplemental video). At one point, the tuna engaged in antipredator behavior presumably to ‘hide’, by swimming very close to the sailfish in front of the video camera along its right flank and out of its peripheral view (Fig. 2f; supplemental video). After roughly 60 s from the tuna’s first appearance on camera, the video and biologging data suggest that the sailfish caught the tuna or terminated the pursuit (Fig. 2b). Because the mouth of the sailfish was not in view of the camera, it is uncertain if the foraging attempt was successful; however, the tuna was last seen directly in front of the sailfish, immediately followed by a headshake (often characteristic of swallowing / prey manipulation for shallowing) and resumption of slow steady swimming by the sailfish, suggesting it was successful (Fig. 2b; supplementary video).

During the 24 h monitoring period, the mean estimated active metabolic rate (AMR_E_; mgO_2_ kg^−1^ h^−1^) of the sailfish for the median, 25th and 75th percentile of all 10,000 iterations were 218.9 ± 70.5, 156.6 ± 48.4 and 307 ± 102.9 mgO_2_ kg^−1^ h^−1^, respectively (Table 2, Figure S5). Using the median iteration, during the dive where the predation occurred, mean AMR_E_ was 518.8 ± 586.3 (IQR 361.2–748.5) mgO_2_ kg^−1^ h^−1^ (Fig. 3). From this median iteration, we estimate that 2.7 MJ (IQR 1.9–3.8 MJ) of energy was expended over the course of the day, where only 1% (0.04 MJ) was expended during the pursuit (Table 2). The estimated energy content of the tuna was 5.1 MJ [calculated from 26], and assuming a successful predation outcome, this encounter resulted in a net energy gain of 2.4 MJ (IQR 1.3–3.2 MJ). However, if this predation was unsuccessful, the cost of this pursuit was only 1% of the energy expenditure for the day. For a sailfish of this size, this daily energy expenditure equates to the required consumption of ~ 0.5 tuna d^−1^ to sustain daily metabolic costs estimated for the median AMR_E_ (218.9 mgO_2_ kg^−1^ h^−1^; see Sect. 3 of supplemental methods for details of calculation).

**Table 2:** Estimated active metabolic rate (AMR_E_; mgO_2_ kg^−1^ h^−1^) and energy expenditure (MJ) during the dive where the predation occurred, and overall for the 24 h period for the 25th, median and 75th percentile of the 10,000 iterations of randomly sampled parameter estimates used to calculate AMR_E_. Values are presented as mean ± SD where applicable.

	Pursuit dive		Overall	
	AMR_E_	MJ	AMR_E_	MJ
25th Percentile	361.2 ± 390.4	0.03	156.6 ± 48.4	1.9
Median	518.8 ± 586.3	0.04	218.9 ± 70.5	2.7
75th Percentile	748.5 ± 874.3	0.06	307 ± 102.9	3.8

**Figure 4:**
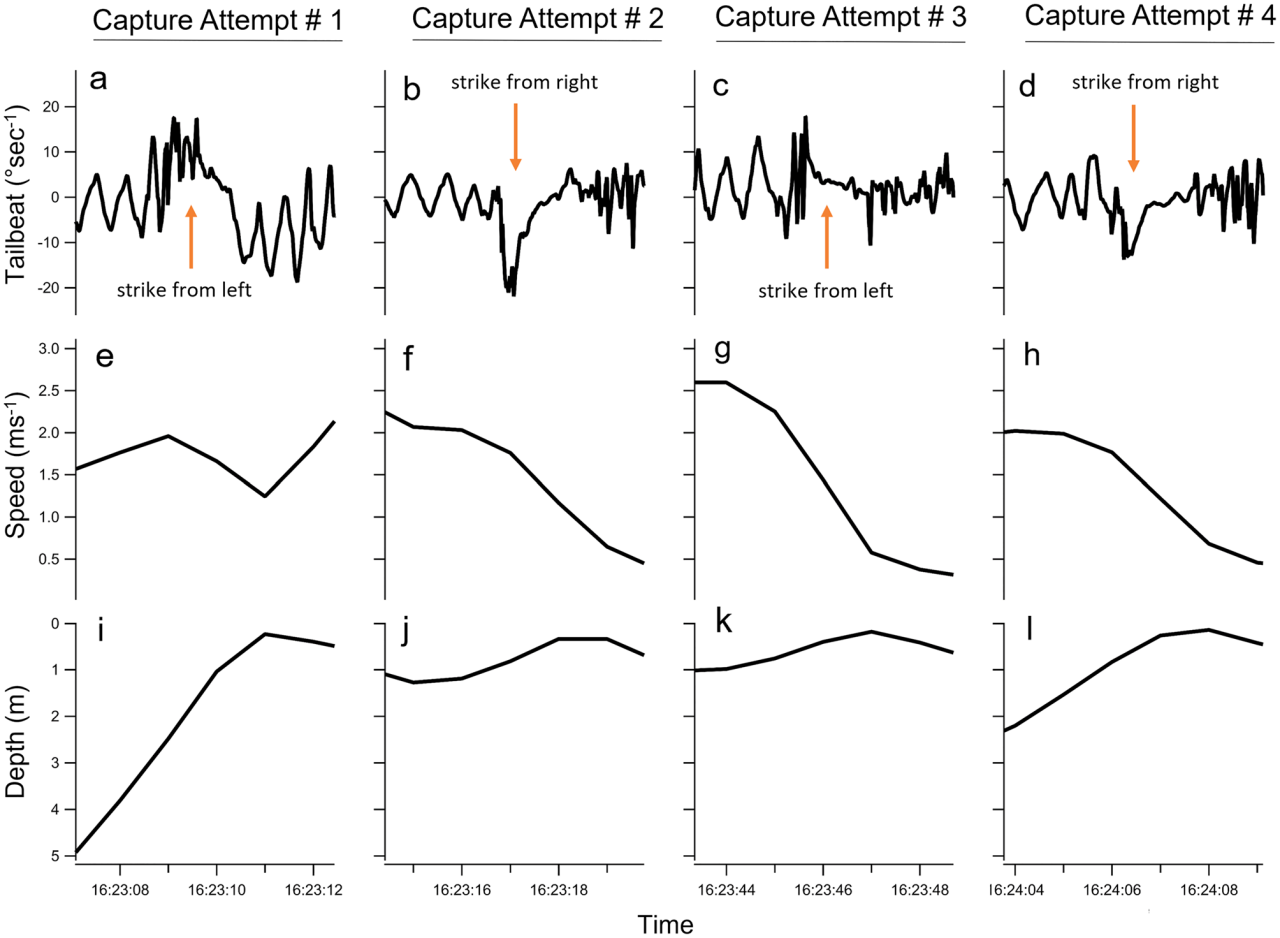
Zoomed in portions (4 s) of the tailbeat (°rotation s^−1^), speed (ms^−1^) and depth (m) of the four different capture attempts (**a**–**d**, respectively) during the one-on-one pursuit (see supplemental video).

We observed a willingness for this individual sailfish to attack from both sides of the prey. The alternating pattern of positive, negative, positive, negative (Fig. 4a–d) in the degrees of rotation s^−1^ immediately prior to and during each strike suggests that the sailfish attacked from different sides of the prey in each successive strike. This behavior is in contrast to Kurvers et al.^8^, who found that during bait-ball hunting aggregations with multiple sailfish, individual sailfish were strongly lateralized and would tend to strike from the same side each time they entered the bait-ball. Attacking from different sides in succession is a novel finding for sailfish and suggests behavioral plasticity within different hunting scenarios (i.e., group vs solitary hunting). In one-on-one hunting situations when neither the predator nor prey are in a group setting, it would benefit the predator to avoid lateralization because the prey can quickly learn any tendencies the predator may have^8,34^.

One noteworthy finding was the relatively low maximum speed attained by the sailfish (3.1 ms^−1^) during the encounter with the prey. Sailfish are believed to be one of the fastest swimming fish^24,27^ with recent estimates suggesting maximum speeds of 8.2–8.3 ± 1.4 ms^−1^^9,28^, and predator–prey interactions might be expected to be events where maximal speeds are exhibited by both predator and prey^9^. However, because it is the prey that sets the speed, timing of accelerations, decelerations and turns, the predator is either reacting to or predicting what the prey will do to enable trajectory interception and capture, which culminates in lower than maximal predator speeds during pursuits^29^. Additionally, theoretical models predict that if prey are slower than their predators, as is the case here^30^, prey should avoid the predator by turning rather than trying to increase separation by travelling as fast as possible^29^. For example, Wilson et al.^29^ demonstrated that if a prey animal is moving as fast as possible, it cannot accelerate forward and must either turn or continue straight, making its movements more predictable and interceptable, compared to a slow moving prey that has more escape options (speed up, slow down, turn) and is therefore less predictable. As such, sailfish and their prey are likely avoiding maximum speeds during one-on-one encounters in open water^31^. Additionally, previous studies have noted that cursorial and avian predators will slow down in the moments prior to an attack to increase maneuverability when in close proximity to prey^29,32,33^, which was also observed here (Fig. 4e–h). Furthermore, morphological adaptations of sailfish (i.e., the bill) can be moved through the water more rapidly than the whole body^9^, potentially allowing sailfish to rely on these morphological ‘weapons’, rather than speed during one-on-one pursuits to increase capture success rates. We also observed a tendency of the sailfish to approach the prey from below in each capture attempt (Fig. 4i–l).

Direct observation of natural predation events by marine predators are rare^35,36^, particularly for pelagic fish predators where visual observation is difficult, prey is sparse, and feeding rates are low compared to that of marine mammals and seabirds^36^. For the predation event presented here, based on the footage of potential prey items near the thermocline (Fig. 2c; supplementary video), the observed increase in speed and TBF immediately prior to the rapid ascent (Figs. 2b; 3a), and the shorter than average dive time (Table 1), we propose that the prey item was encountered at depth, and chased to the surface^6^. Given the shallow thermocline and co-occurring oxycline present in the Eastern Tropical Pacific^37^, and the potential for these features to concentrate prey^38–40^, we hypothesize that oscillatory dives in the mixed layer are prey-searching dives to increase foraging opportunities^41^. Due to their unique metabolic biochemistry suited to life in the open ocean, tunas and billfishes have long been described as energy speculators, gambling high and continual energy output, expecting to offset the costs with periodic high energetic gain events^42–44^. The estimated energy gain of 2.4 MJ resulting from the prey encounter in the 24 h period described here is consistent with energy speculation behavior, but also suggests this sailfish would need to regularly forage on high energy prey to support its metabolic requirements. Because the video camera was only recording for the daylight hours of the 24 h period analyzed here, we cannot say if any other feeding events occurred during the remainder of the track; however, there were two other similar bursts of activity identified in the acceleration data during the following day prior to the tag releasing from the fish. As such, energy obtained from individual foraging events like that described here may be an important energy supply for the routine energy requirements of these high metabolic performers between opportunistic, large energy gain, group hunting bait-ball foraging events where multiple prey can be consumed in a short amount of time.

## Conclusions

This biologger deployment yielded animal-borne video, triaxial acceleration, gyroscope and magnetometer data, and depth, temperature, and speed data of a rare predation event, along with a full 24 h period of behavioral and activity data providing a one-of-a-kind dataset for this top predator. We estimated a range of values in which this sailfish’s active metabolic rate likely occurred (IQR 156 ± 48 to 307 ± 102, median 219 ± 70; mgO_2_ kg^−1^ h^−1^ at 0.21 ± 0.1 BLs^−1^; Table 2), and cursory interspecific comparisons suggest our estimates of metabolic rate fall within the range of expected values^45–48^. However, it should be noted that because dolphinfish were used as the proxy species and they do not possess cranial endothermy as sailfish do, the estimates presented here are likely an underestimation. Furthermore, the measurements presented here are based on a single sailfish, and individual variability in diving behavior would impact estimated metabolic costs. Undoubtedly, as new methods are developed to directly quantify oxygen consumption of large fishes [e.g., 48], estimates of metabolic rate will become more accurate and will enable estimates for additional physiological measurements, such as aerobic scope and cost of transport. However, we provide the first detailed description of activity levels and hunting behavior of an individual free-ranging sailfish and estimate the energetics of a rare predation event. These estimates suggest that while the energetic gains from this predation event were substantial compared to what was expended during the pursuit, the amount of energy burned in search of prey over the course of the day and night was considerable. Until methods to directly quantify metabolic costs of large pelagic predators are developed, the approach we have taken could be used as a starting point to inform future energetic and trophic models and improve our understanding of the role of these pelagic predators in our oceans.

## Methods

The sailfish (estimated to be 40 kg by an experienced captain, and calculated to be 1.85 m per Wares and Sakagawa^50^) was caught via rod and reel from a recreational sportfishing vessel using standard trolling gear with natural bait off the Pacific coast of southeast Panama (7.53 N, 78.53 W). The fish was brought alongside the vessel and a custom-designed biologging tag package was attached to the dorsal musculature with two umbrella dart anchors (Fig. 1). Once both anchors were securely imbedded in the muscle, the tag was cinched against the body using two galvanic timed releases (International Fishing Devices Inc., Northland, New Zealand) and a cable tie. Only 6 min elapsed from when the fish was hooked to release. The tag consisted of an acceleration data logger (tri-axial accelerometer, magnetometer and gyroscope recording at 100 Hz), depth and temperature sensors, and a small turbine-based fluid speed sensor recording at 1 Hz (OpenTag 3.0, Loggerhead Instruments, Sarasota, FL; see supplemental data for swim speed calibration; Figure S1, S2). Finally, the tag package contained a miniaturized video camera (68 mm × 21 mm × 22 mm; Little Leonardo, Tokyo, Japan) and a Smart Position and Temperature tag (SPOT-363A; Wildlife Computers, Redland WA) to aid in package recovery. The entire tag package was 18 × 7 cm at the leading edge, increasing to 18 × 10.5 cm at the trailing edge, weighing 335 g in air (~ 0.8% of sailfish body weight; 4–10% of the frontal cross-sectional area of the sailfish; see supplemental methods for estimated drag). Upon dissolution of the galvanic timed releases, the package released from the fish and was recovered at sea using a UHF handheld receiver (AOR AR8200, USA).

Data were analyzed using Igor Pro v. 8.0.4.2 (Wavemetrics, Inc., Lake Oswego, OR, USA) and RStudio v. 1.4.1106^51^. The static component of acceleration was calculated using a 3-s box smoothing window on the raw acceleration data as this was visually determined to sufficiently remove the dynamic component of acceleration^52^. Tag attachment angle was corrected by rotating the raw acceleration data such that the X and Y axis had a mean of zero. Body pitch was then calculated from the anterior–posterior axis of the static component of acceleration. The lateral axis of the gyroscope was used to determine directionality of the strikes during the predation event, and calculate the tailbeat frequency using a continuous wavelet transformation^35,53^. Finally, a compass heading and reconstructed track were generated from the magnetometer data using the *magHead* function in the gRumble R package^54^.

To estimate the sailfish’s active metabolic rate and energy expenditure, we used the relationship between oxygen consumption and swim speed for adult dolphinfish (*Coryphaena hippurus*)^55^, with the assumption this relationship is consistent across fish length^56,57^. See Sect.  2 of the supplementary material for a detailed description of why dolphinfish was chosen as the proxy species and further description of metabolic rate calculations. Oxygen consumption (*M*O_2_; mgO_2_ kg^−1^ h^−1^) was estimated using the equation log(*M*O_2_) = [c*U* + log(d)], where c and d are the slope and intercept of the logarithmic regression, and *U* is the swim speed of the sailfish (BLs^−1^; Figure S3). *M*O_2_ was calculated continuously for every speed measurement throughout the 24 h from the sailfish tag data, and we then took the inverse log of *M*O_2_ and corrected for mass of the dolphinfish (M_D_) in ^55^ to obtain *V*O_2_ (mgO_2_ h^−1^). Oxygen consumption for the 40 kg sailfish was then calculated using the equation:

$${\text{AMR}}_{{\text{E}}} = V{\text{O}}_{2} \left( { \frac{{{\text{M}}_{{\text{S}}} }}{{{\text{M}}_{{\text{D}}} }}} \right)^{{\text{b}}}$$

where AMR_E_ is the estimated active metabolic rate (mgO_2_ h^−1^), *V*O_2_ is the oxygen consumption at each swim speed, b is the mass scaling exponent, and M_S_ is the sailfish mass (kg). AMR_E_ was corrected for temperature using a Q_10_ of 1.83 ^58,59^ and was made mass-specific using the estimated mass of the sailfish (see Sect. 2 of the supplementary information for more detail).

Because a proxy species was used for the calculation, we allowed for variation in parameter estimates of b, c, d, and M_D_ with an iterative approach (10,000 iterations) and randomly sampled values for these parameters from normal distributions with means and standard deviations equal to published values where available (Table S1, Figure S4). The median of all iterations was used as the AMR_E_, with the interquartile range (IQR) used to represent a range of possible AMR_E_ values (Figure S5). Sailfish swim speed (ms^−1^) was converted to BL s^−1^ after estimating the length using previously published length—mass relationships^50^. To calculate energy expenditure of the day and the predation event, we used an oxy caloric coefficient of 0.013 kJ mgO_2_^−1^ ^60^, and 8.03 kJ g^−1^ wet weight for *Auxis* spp*.* energy content^26^. Prey mass of 635 g was estimated for a 35 cm TL tuna using the length–weight relationship for *A. thazard* ^61^.

### Ethical approval and informed consent

The sailfish was tagged under permit from the Ministerio de Ambiente, República de Panamá (SE/A-64-19). All procedures were approved by Nova Southeastern University’s Institutional Animal Care and Use Committee (2019.04.MS1). All methods were performed in accordance with the relevant guidelines and regulations. The study is reported in accordance with ARRIVE guidelines.

## Supplementary Information

Supplementary Information 1.

Supplementary Video S1.

Supplementary Information 2.

## Data Availability

The dataset is available and will be made public upon acceptance at the dryad data repository DOI https://doi.org/10.5061/dryad.vdncjsxzb. For peer review, the available dataset can be accessed here: https://datadryad.org/stash/share/Pa6OgGCg09sQvgkypSLs6Bc_AeNlUalD4uxMlqNqA0Q.
