# Supplementary material for: Hunting behavior of a solitary sailfish Istiophorus platypterus and estimated energy gain after prey capture
Source: Sci Rep. 2023 Jan 27;13:1484. doi: 10.1038/s41598-023-28748-0 (PMC9883507; doi:10.1038/s41598-023-28748-0)
Supplement: Supplementary file 1 — Supplementary Information 1. [file 41598_2023_28748_MOESM1_ESM.pdf]

Supplemental Information to: Hunting behavior of a solitary sailfish *Istiophorus platypterus* and estimated energy gain after prey capture

Ryan K. Logan[1], Sarah M. Luongo[2], Jeremy J. Vaudo[1], Bradley M. Wetherbee[1,3], Mahmood S. Shivji[1]

[1]Guy Harvey Research Institute, Nova Southeastern University, Dania Beach, FL USA
[2]Department of Biological Sciences, Florida International University, North Miami, FL USA
[3]Department of Biological Sciences, University of Rhode Island, Kingston, RI USA

Supplemental Methods and Data Analysis

1. Swim Speed and Drag

To calibrate the turbine speed sensor with water velocity, the completed tag package was placed into a 90-L Loligo swim tunnel respirometer with a flow-meter, resulting in a measured linear relationship of m/s = 0.022 * rotations/s + 0.25 ($r^2 = 0.99$, $p < 0.0001$; Fig. S1). It was found that the turbine requires a minimum flow speed of ~ 0.25 m/s to turn, therefore, after the calibration step any calculated speed of $\leq$ 0.25 m/s was set to 0.25 m/s to not underestimate.

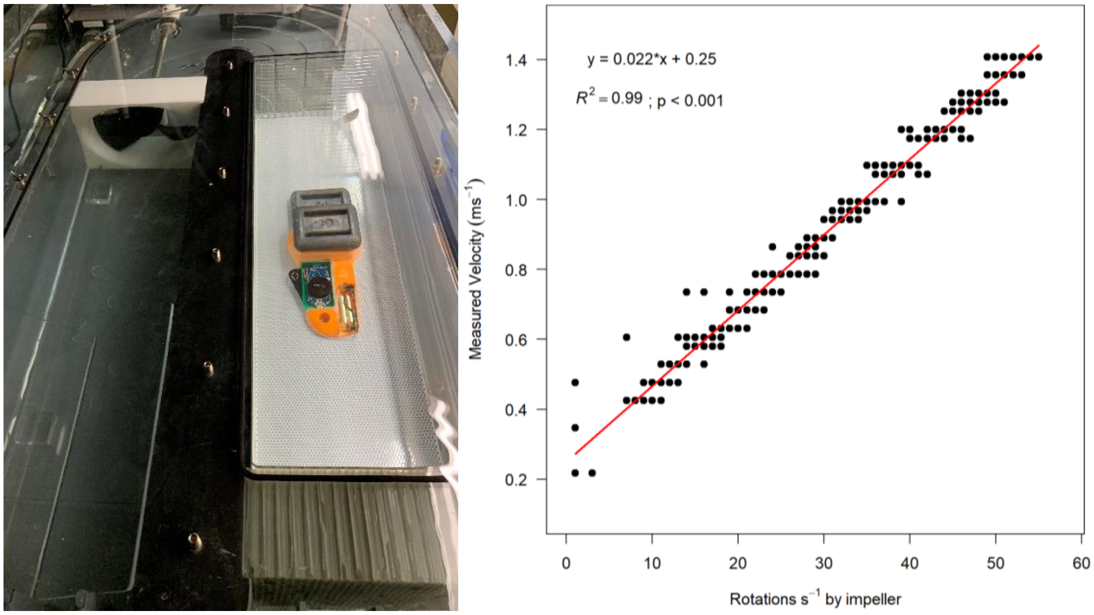

Figure S1. Completed biologging tag package in 90 L Loligo swim tunnel, and associated linear regression of swim speed calibration.

While this calibration step in the flume was necessary, these are ideal conditions and may not be representative of the situation once it is attached to a fish. To ensure that the speed sensor in the flume accurately represents the velocity once it is attached to a fish, we compared the speed measured by the impeller to another established method of calculating speed via the vertical velocity of the fish (ms^{-1}) and the body pitch angle derived from the accelerometer, using body pitch angles of > 20° [1, 2]. We regressed the vertical velocity (m/s) and body pitch angle method against the speed measured by the impeller attached to the tag. The blue line is the linear regression of the two, and the black line represents a 1:1 relationship. In general, the tag speed sensor matches the calculated speed method with some variability ($p < 0.001$, $r^2 = 0.7$, $y = 1.03x – 0.02$). The advantage of using the impeller is we are still able to obtain a speed measurement at low body pitch angles or at times when the fish is at a constant depth.

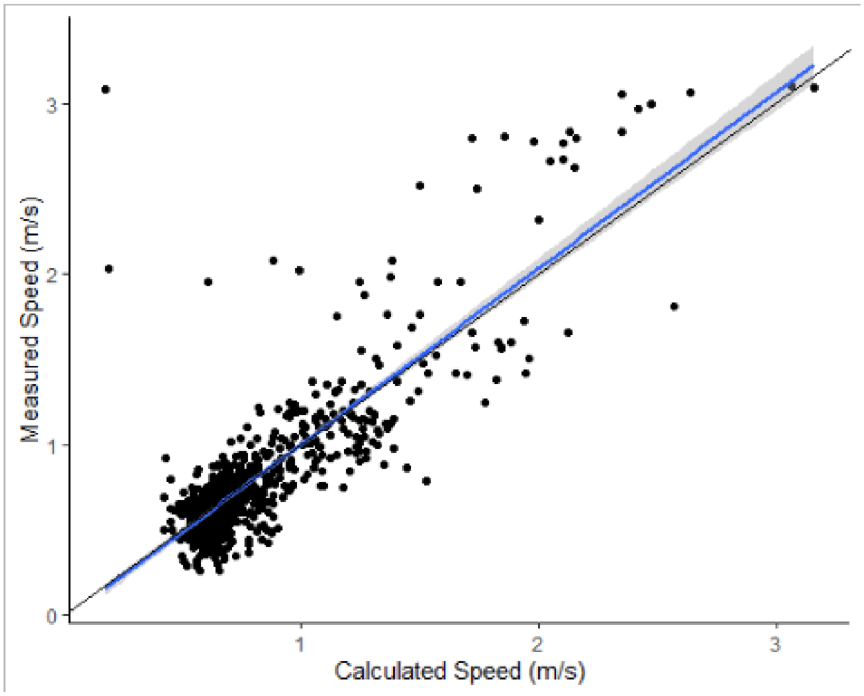

Figure S2. Linear regression (blue line) of the speed measured by the animal-borne impeller (Measured Speed (m/s)) and the calculated speed using the equation speed (m/s) = vertical

velocity (m/s) / sin(φ), where φ is the body pitch. $p < 0.001$, $r^2 = 0.7$, $y = 1.03x - 0.02$. The black line represents a 1:1 relationship.

Finally, while there is little doubt that the tag had some effect on the drag of the sailfish, in the absence of swim tunnel experiments with and without a tag, we cannot say with certainty exactly how much, and how this may have affected the sailfish's behavior. However, using cross-sectional area measurements from the largest section of a similarly sized sailfish (just posterior to the head; where the tag was placed) [3] we calculated the drag acting on a body of that area using the equation $F_D = 0.5C\rho Av^2$, where C is the drag coefficient (0.24 [unitless]) [3], ρ is the density of seawater at 27°C [1023 kg m^{-3}], A is the area of the object (cross-sectional area; 0.025 m^2), and ν is the velocity of the object (1 m/s). This resulted in a drag of 3.1 N acting on the sailfish. Because the tag was designed to be as hydrodynamic as possible, it is narrower at the leading edge (facing in the direction of travel) and gets thicker at the trailing edge (seen in figure 1 and figure S1). Using the cross-sectional area from the narrow end of the tag (0.001 m^2) and a drag coefficient of 0.6, we calculated an added drag of 0.3 N, or 9% of the drag on the sailfish. Using the largest portion of the tag for the cross-sectional area (0.0026 m^2), we calculated a drag of 0.79 N, or roughly 25% of the drag acting on the sailfish. While we cannot say exactly how this may have impacted behavior in the absence of behavioral data without a tag, Sagong et al. (2013) found that there was a 21.5% increase in the drag when they attached pectoral fins to their specimens. As such, a 9-25% increase from the tag does not appear to be a major increase, but there is undoubtedly some effect of tag attachment which may lead to an underestimation of metabolic rate estimates.

2. Proxy Species Selection & Metabolic Rate Calculation

Obtaining direct measurements of oxygen consumption at varying mass, swim speeds and activity levels is not currently feasible for sailfish. However, recent studies indicate that lifestyle,

trophic level, and morphology are correlated with metabolic rate such that pelagic, upper trophic level fishes with similar morphology (e.g. high caudal fin aspect ratio, gill surface area) exhibit similar and elevated metabolic rates [4-7]. As such, sailfish may have metabolic demands comparable to dolphinfish [*Coryphaena hippurus*; 8], another subtropical epipelagic predator with comparable ecological interactions [9]. Additionally, the gill surface area to body mass ratio is very similar between dolphinfish and the closely related striped marlin (*Kajikia audax*) [12], indicating similar oxygen uptake capabilities between dolphinfish and istiophorid billfishes regardless of body size, further suggesting dolphinfish provide a suitable proxy for sailfish metabolic rate. Although sailfish possess cranial endothermy and warm their brain and retina with a specialized thermogenic organ that sits beneath the brain, the rest of the body is ectothermic and does not retain metabolic heat [10]. Therefore, we did not feel that a subtropical scombrid with endothermy, such as yellowfin tuna (*Thunnus albacares*), which warm their muscle, viscera and brain via vascular counter-current heat exchangers, were an acceptable proxy species for this study [11]. Although dolphinfish are smaller than the sailfish tagged in the present study, the effects of body size on swimming metabolic rates in fishes can be removed by using swim speed relative to body length [13]. More specifically, log swimming metabolic rates plotted against swim speed relative to body length produce similar straight lines independent of body size of the fish [13]. Owing to the lack of direct measurements of swimming metabolic rates for larger fishes with regional endothermy, data for dolphinfish was regarded as the best available information. Therefore, we took the equation of the line ($y = 0.1168x^2 – 0.6457x + 1.1994$) used to describe the relationship between the cost of transport ($mgO_2\ kg^{-1}\ m^{-1}$) and swim speed (U; $BL\ s^{-1}$) of dolphinfish in the control group (no oil exposure) of [14] . We then converted cost of transport to mass-specific oxygen consumption ($\dot{M}O_2$, $mgO_2\ kg^{-1}\ h^{-1}$) by

multiplying by both the mean fork length of dolphinfish in the control group (0.291 m) and 3600 s. Oxygen consumption (MO_2; $mgO_2\ kg^{-1}\ h^{-1}$) at various swim speeds was estimated using the equation $\log(MO_2) = [cU + \log(d)]$, where c and d are the slope and intercept of the logarithmic regression, and U is the swim speed (BLs^{-1}) after correcting for the BL of the sailfish (Figure S3). By correcting for the body length of the sailfish, the range of BLs^{-1} for the sailfish spans 0.08-0.63 BLs^{-1}, accounting for the majority of our observed speed data, and we linearly extrapolate to higher swim speeds (Figure S3). MO_2 was calculated continuously for every speed measurement throughout the 24 hours from the sailfish tag data, and we then took the inverse log of MO_2 and corrected for mass of the dolphinfish (M_D) in [14] to obtain VO_2 ($mgO_2\ h^{-1}$). Oxygen consumption for the 40 kg sailfish was then calculated using the equation:

$$\mathrm{AMR_E} = VO_2\left(\frac{\mathrm{M_S}}{\mathrm{M_D}}\right)^{\mathrm{b}}$$

where AMR_E is the estimated active metabolic rate ($mgO_2\ h^{-1}$), VO_2 is the oxygen consumption at each swim speed ($mgO_2\ h^{-1}$), b is the mass scaling exponent, and M_S is the sailfish mass (kg). We corrected AMR_E for temperature by multiplying by Q_{10}^$((T_2-T_1)/10)$ [16], where Q_{10} is the increase in standard metabolism with an increase in 10°C, T_1 is the temperature the dolphinfish were tested at in [14], and T_2 was the continuous temperature experienced by the sailfish, with Q_{10} set to 1.83 [5, 15]. AMR_E was then made mass-specific to the estimated mass of the sailfish and corrected to units of $mgO_2\ kg^{-1}\ h^{-1}$.

3. Energy Expenditure and Prey Consumption

To estimate the amount of energy expended over the course of the 24-h period and during the predation event, we first converted the AMR_E from $mgO_2\ kg^{-1}\ h^{-1}$ to $kJ\ kg^{-1}\ h^{-1}$ by multiplying by the oxy caloric coefficient of 0.013 $kJ\ mgO_2^{-1}$. Because we sum across seconds, we then divide this by 3600 leaving $kJ\ kg^{-1}\ sec^{-1}$, and finally multiplied by the estimated mass of the

sailfish (40 kg), leaving an estimate of kJ burned sec^{-1} for the sailfish. Each value of kJ sec^{-1} was then summed across each period (the entire day or just the predation event; Table 2 of the main text) to determine energy expended. Finally, to estimate the daily amount of prey needed to maintain metabolic costs, we used the mean AMR_E of the 24 h period, converted it from mgO_2 kg^{-1} h^{-1} to kJ kg^{-1} h^{-1}, then to kJ. This value was then divided by the estimated energy content of the tuna (5.1 kJ), to arrive at 0.5 tuna d^{-1} to sustain daily AMR_E.

Supplemental Tables

Table S1. Mean ± SD of the normal distributions used when randomly sampling parameter values for b, c, d and dolphinfish mass (M_D) used in the 10,000 iterations when calculating the range of possible values of estimated active metabolic rate (AMR_E) of the sailfish.

Parameter	Value	Reference
b	0.79 ± 0.1	[5, 15, 17, 18]
c	0.9 ± 0.09	Estimated from regression of log(MO_2) ~ sailfish swim speed, back calculated from [14]
d	2.6 ± 0.04	Estimated from regression of log(MO_2) ~ sailfish swim speed, back calculated from [14]
M_D (g)	278 ± 23	[14]

Table S2. Estimated metabolic rate for the 25th percentile, median and 75th percentile during the pursuit dive with and without applying a temperature correction to the calculations.

With Temperature Correction			Without Temperature Correction		
	AMR_E (mgO2/kg/h)	Energy Expenditure		AMR_E (mgO2/kg/h)	Energy Expenditure
25th percentile	361 ± 390	0.02	25th percentile	429 ± 514	0.03
Median	518 ± 586	0.04	Median	618 ± 773	0.05

75th percentile	748 ± 874	0.06	75th percentile	894 ± 1153	0.07

Table S3. Overall estimated metabolic rate for the 25th percentile, median and 75th percentile over the course of 24 hours with and without applying a temperature correction to the calculations.

With Temperature Correction			Without Temperature Correction		
	AMR_E (mgO2/kg/h)	Energy Expenditure		AMR_E (mgO2/kg/h)	Energy Expenditure
25th percentile	156 ± 48	1.9	25th percentile	157 ± 58	1.9
Median	219 ± 70	2.7	Median	220 ± 85	2.7
75th percentile	307 ± 102	3.8	75th percentile	308 ± 125	3.9

Additional Supplemental Figures

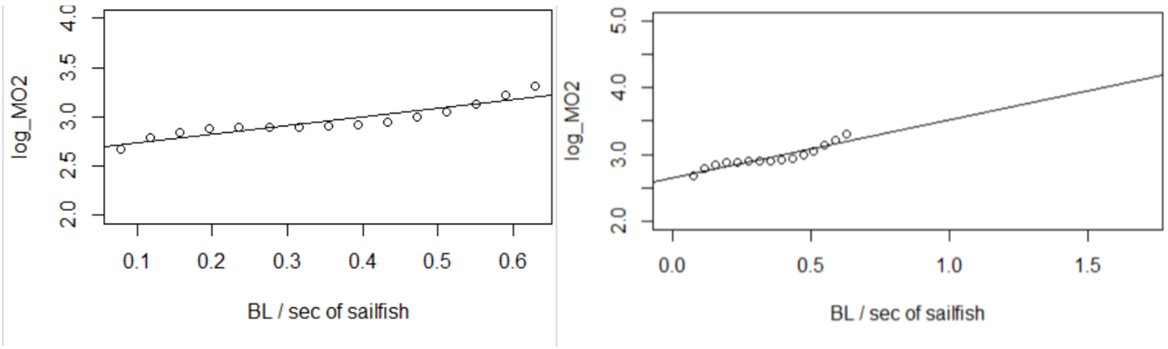

Figure S3. Logarithmic regression of dolphinfish (*Coryphaena hippurus*) oxygen consumption (MO_2), back-calculated from [14] and swimming speed (body length [BL] s^{-1}), corrected for BL of the sailfish. Measured values correspond to those measured in [14] (1-4 BLs^{-1} of dolphinfish = 0.08 – 0.63 BLs^{-1} of sailfish; left), and linearly extrapolated over the range of swimming speeds observed from the sailfish tagged in the present study (right).

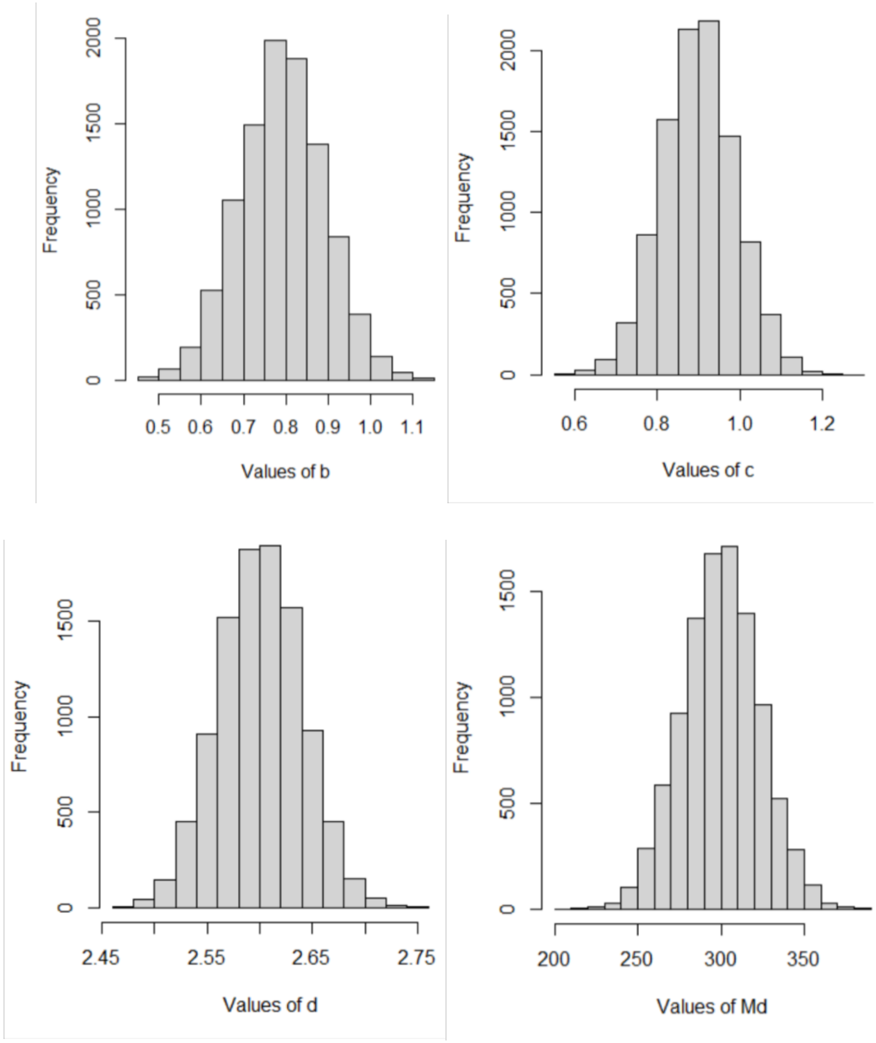

Figure S4. Histograms displaying the possible values of b, c, d and M_D used in the 10,000 iterations when calculating the estimated active metabolic rate (AMR_E) of the sailfish.

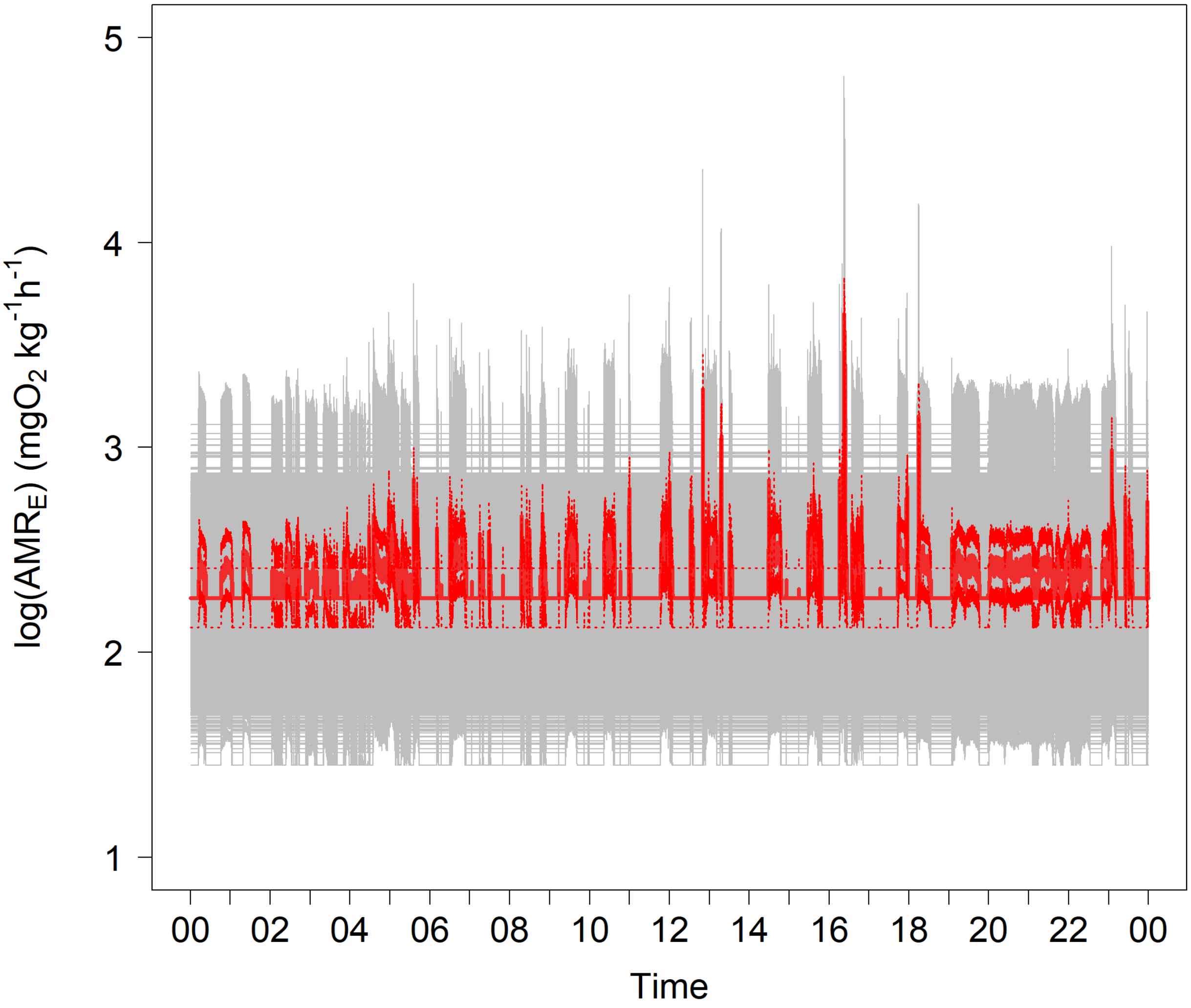

Figure S5. Output of the log transformed estimated active metabolic rate (AMR_E; $mgO_2\ kg^{-1}\ h^{-1}$) from the 10,000 iterations over the 24 h period. AMR_E was calculated as the median of the 10,000 samples (solid red line), with the interquartile range (25 - 75%) taken to represent a range of probable AMR_E values (dotted red lines).

References

1. Gleiss, A.C., B. Norman, and R.P. Wilson, *Moved by that sinking feeling: variable diving geometry underlies movement strategies in whale sharks.* Functional Ecology, 2011. **25**(3): p. 595-607.
2. Andrzejaczek, S., et al., *Depth-dependent dive kinematics suggest cost-efficient foraging strategies by tiger sharks.* Royal Society open science, 2020. **7**(8): p. 200789.

3. Sagong, W., W.-P. Jeon, and H. Choi, *Hydrodynamic characteristics of the sailfish (Istiophorus platypterus) and swordfish (Xiphias gladius) in gliding postures at their cruise speeds.* PloS one, 2013. **8**(12): p. e81323.
4. Killen, S.S., et al., *Ecological Influences and Morphological Correlates of Resting and Maximal Metabolic Rates across Teleost Fish Species.* The American Naturalist, 2016. **187**(5): p. 592-606.
5. Killen, S.S., D. Atkinson, and D.S. Glazier, *The intraspecific scaling of metabolic rate with body mass in fishes depends on lifestyle and temperature.* Ecology letters, 2010. **13**(2): p. 184-193.
6. Norin, T. and B. Speers-Roesch, *Metabolism*, in *The Physiology of Fishes*, S. Currie and D.H. Evans, Editors. 2021, CRC Press: Boca Raton, FL. p. 129 - 141.
7. Bigman, J.S., et al., *Respiratory capacity is twice as important as temperature in explaining patterns of metabolic rate across the vertebrate tree of life.* Science Advances, 2021. **7**(19): p. eabe5163.
8. Brill, R.W., *Selective advantages conferred by the high performance physiology of tunas, billfishes, and dolphin fish.* Comparative Biochemistry and Physiology Part A: Physiology, 1996. **113**(1): p. 3-15.
9. Amezcua Gómez, C.A., *Relaciones tróficas entre el pez vela (Istiophorus platypterus) y el dorado (Coryphaena hippurus) en la costa de los estados de Jalisco y Colima, México*. 2007, Instituto Politécnico Nacional. Centro Interdisciplinario de Ciencias Marinas. p. 110.
10. Block, B.A., *Structure of the brain and eye heater tissue in marlins, sailfish, and spearfishes.* Journal of Morphology, 1986. **190**(2): p. 169-189.
11. Block, B.A. and J.R. Finnerty, *Endothermy in fishes: a phylogenetic analysis of constraints, predispositions, and selection pressures.* Environmental Biology of Fishes, 1994. **40**(3): p. 283-302.
12. Wegner, N.C., et al., *Gill morphometrics in relation to gas transfer and ram ventilation in high-energy demand teleosts: Scombrids and billfishes.* Journal of Morphology, 2010. **271**(1): p. 36-49.
13. Beamish, F., *Swimming capacity. In 'Fish Physiology. Vol. VII'.(Eds WS Hoar and DJ Randall.) pp. 101–187*. 1978, Academic Press: New York.
14. Stieglitz, J.D., et al., *Impacts of Deepwater Horizon crude oil exposure on adult mahi-mahi (Coryphaena hippurus) swim performance.* Environmental Toxicology and Chemistry, 2016. **35**(10): p. 2613-2622.
15. Clarke, A. and N.M. Johnston, *Scaling of metabolic rate with body mass and temperature in teleost fish.* Journal of animal ecology, 1999. **68**(5): p. 893-905.
16. Schmidt-Nielsen, K., *Animal physiology: adaptation and environment*. 1997: Cambridge university press.
17. Brodie, S., et al., *Improving consumption rate estimates by incorporating wild activity into a bioenergetics model.* Ecology and Evolution, 2016. **6**(8): p. 2262-2274.
18. Payne, N.L., et al., *A new method for resolving uncertainty of energy requirements in large water breathers: the 'mega-flume'seagoing swim-tunnel respirometer.* Methods in Ecology and Evolution, 2015. **6**(6): p. 668-677.
