# Supplementary material for: Hunting behavior of a solitary sailfish Istiophorus platypterus and estimated energy gain after prey capture
Source: Sci Rep. 2023 Jan 27;13:1484. doi: 10.1038/s41598-023-28748-0 (PMC9883507; doi:10.1038/s41598-023-28748-0)
Supplement: Supplementary file 3 — Supplementary Information 2. [file 41598_2023_28748_MOESM3_ESM.docx]

Supplemental video 1. Point-of-view video of the sailfish predation event. See figure 2a for tag placement. The sailfish rapidly ascends from ~ 60 m depth to the surface, where the prey first becomes visible. The sailfish makes several attempts to capture the prey, ultimately culminating in what is assumed to be a successful capture. The majority of the video is real time, but portions have been slowed down or momentarily stopped to visualize the prey fish, with a red circle outlining the prey.
